# Effectiveness of a universal parental support programme to promote health behaviours and prevent overweight and obesity in 6-year-old children in disadvantaged areas, the Healthy School Start Study II, a cluster-randomised controlled trial

**DOI:** 10.1186/s12966-016-0327-4

**Published:** 2016-01-21

**Authors:** Gisela Nyberg, Åsa Norman, Elinor Sundblom, Zangin Zeebari, Liselotte Schäfer Elinder

**Affiliations:** Department of Public Health Sciences, Karolinska Institutet, Tomtebodavägen 18 A, 171 77 Stockholm, Sweden; Centre for Epidemiology and Community Medicine, Stockholm County Council, Box 1497, 171 29 Solna, Sweden

**Keywords:** Physical activity, Diet, Sedentary behaviour, Accelerometer, BMI, Intervention, Socio-economic status, Parental education, Motivational interviewing, Pre-school class

## Abstract

**Background:**

There is increasing evidence for the effectiveness of parental support programmes to promote healthy behaviours and prevent obesity in children, but only few studies have been conducted among groups with low socio-economic status. The aim of this study was to develop and evaluate the effectiveness of a parental support programme to promote healthy dietary and physical activity habits and to prevent overweight and obesity in six-year-old children in disadvantaged areas.

**Methods:**

A cluster-randomised controlled trial was carried out in disadvantaged areas in Stockholm. Participants were six-year-old children (*n* = 378) and their parents. Thirty-one school classes from 13 schools were randomly assigned to intervention (*n* = 16) and control groups (*n* = 15). The intervention lasted for 6 months and included: 1) Health information for parents, 2) Motivational Interviewing with parents and 3) Teacher-led classroom activities with children. Physical activity was measured by accelerometry, dietary intake and screen time with a questionnaire, body weight and height were measured and BMI standard deviation score was calculated. Measurements were conducted at baseline, post-intervention and at 5months follow-up. Group effects were examined using Mixed-effect Regression analyses adjusted for sex, parental education and baseline values.

**Results:**

Fidelity to all three intervention components was satisfactory. Significant intervention effects were found regarding consumption of unhealthy foods (*p* = 0.01) and unhealthy drinks (*p* = 0.01). At follow-up, the effect on intake of unhealthy foods was sustained for boys (*p* = 0.03). There was no intervention effect on physical activity. Further, the intervention had no apparent effect on BMI sds for the whole sample, but a significant difference between groups was detected among children who were obese at baseline (*p* = 0.03) which was not sustained at follow-up.

**Conclusions:**

The Healthy School Start study shows that it is possible to influence intake of unhealthy foods and drinks and weight development in obese children by providing individual parental support in a school context. However, the effects were short-lived. Therefore, the programme needs to be prolonged and/or intensified in order to obtain stronger and sustainable effects. This study is an important contribution to the further development of evidence-based parental support programmes to prevent overweight and obesity in children in disadvantaged areas.

## Background

Insufficient physical activity and poor dietary habits are important lifestyle factors causing chronic diseases worldwide, including obesity [1]. Studies show that health-related behaviours [2–4] and obesity [5] track from childhood to adolescence and adulthood. This might lead to serious health consequences later in life, such as metabolic disturbances, type-2 diabetes, cardiovascular diseases, certain cancers and impaired mobility [6]. Furthermore, children of obese parents often develop a similar weight pattern, and low socio-economic status (SES) is one of the strongest determinants [7].

In Sweden, as in many high income countries, there are large social inequalities in dietary habits, physical activity and prevalence of obesity to the disadvantage of children from families with low SES [8, 9]. Children living in deprived areas have approximately three times greater risk of becoming obese than children living in affluent areas [8, 10], pointing to the importance of social factors. Therefore, interventions targeting health-related behaviours and obesity should focus on both social and environmental determinants and start from an early age. Parents and factors in the home environment are important for children’s dietary habits [11–14] and physical activity [15–18]. Moreover, lower SES is associated with poorer eating habits [19]. A Swedish study shows that already from grade 2 (age 8), there are clear differences in eating habits depending on the parents’ educational background [20]. In contrast, most studies done among young children, find no association between parental SES and physical activity [16, 21].

In order to reach all children, irrespective of family background, schools are the preferred setting for health promotion. The effectiveness of school-based programmes can be enhanced by including a parental component [22]. In recent years, evidence has been accumulating for parental support programmes to promote healthy dietary and physical activity habits in school children [23–25]. Furthermore, a number of successful parental support programmes with [26] or without a school component suggest greater effectiveness the lower the age of the participating child [27]. A review about parental involvement in efforts to improve children’s diet concluded that most interventions used indirect methods such as sending home newsletters, but that direct approaches such as group education were more likely to be effective [24]. A review of parental support interventions targeting children’s health behaviours showed that individual counselling with parents was effective in improving children’s dietary habits but less effective in increasing physical activity [27]. The review also showed that in minority groups and groups with low SES, intensive parental support given in group educational settings is promising, but low participation and attrition remain a challenge. Moreover, few studies had used an individual counselling approach with parents in disadvantaged groups.

Young children have a limited cognitive capacity for decision making and therefore rely on care-givers. It has been suggested that parental self-efficacy (PSE) impacts child behaviour both directly and indirectly via parenting practices and behaviours, and that PSE may be an appropriate target for intervention [28]. One way to improve PSE could be through Motivational Interviewing (MI), a method used to support behaviour change [29]. There is evidence that MI may improve dietary and physical activity habits in adults and enhance weight loss in overweight and obese patients [30–32]. Little is yet known of the effectiveness of MI as a way of influencing parents to improve health related behaviours of their children, but studies suggest that MI may increase parents’ understanding of their children’s weight problems and their motivation to improve their children’s health habits [33, 34]. MI has also been suggested as a strategy to support constructive parenting skills in general [35].

The aim of this study was to evaluate the effectiveness of the 6-month Healthy School Start parental support programme targeting dietary habits, physical activity and body weight of six-year-old children in families with low socio-economic status in the school context.

## Methods

### Study design, randomisation, setting and participants

The intervention A Healthy School Start has been evaluated previously in an area with low to medium socio-economic status [36, 37]. Based on experiences from the first study, we designed this second study as a cluster-randomised controlled trial with waiting-list control group. The unit of randomisation was pre-school class. Schools were chosen from low income areas in a municipality in Stockholm County, Sweden, with the highest prevalence of overweight and obesity among children in the county [38]. These areas are characterised by a high proportion of foreign-born citizens. This study involved 378 six-year-old children in pre-school class.

**Fig. 1:**
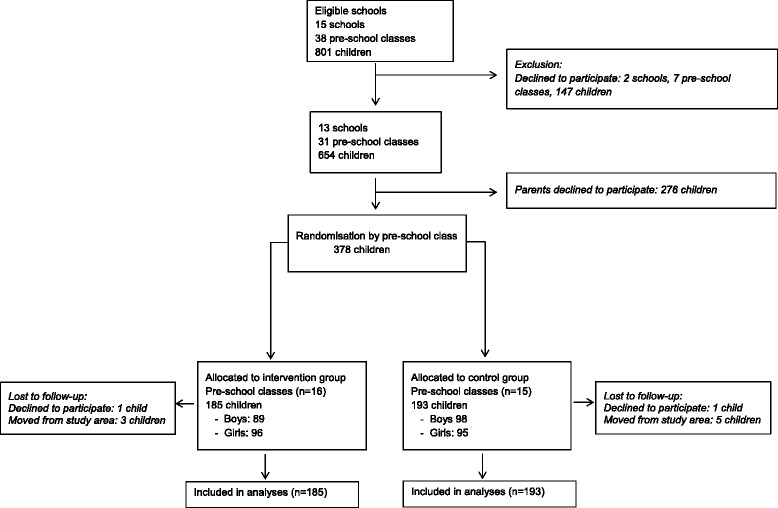
Flow diagram of participants

Figure 1 shows participant recruitment and retention. Of the 15 eligible schools (*n* = 801 children) in three low-income areas, 13 schools and 31 pre-school classes participated. All families who had children in these classes were invited to participate in the study. The classes were randomly assigned to intervention (*n* = 16) or waiting-list control group (*n* = 15) after baseline measurement. Each class was assigned a number which was drawn randomly from a basket by an independent person in the presence of the research team. Every other school class was assigned to the intervention group. The children were recruited in August to September 2012, the intervention started in October and lasted for six months (2012–2013). Pre-school class is not compulsory in Sweden but 90–95 % of all six-year-old children attend.

Written consent was collected from all parents of participating children. The study was approved by the Regional Ethical Review Board in Stockholm, Sweden (2012/877–31/5) on the 14^th^ of June, 2012. The trial has been registered: ISRCTN39690370.

### Theory

It is widely recommended that programme design should be based on theory [39] where a theoretical framework supports the identification of the causal chain and possible mediators. Social cognitive theory (SCT) explains behaviour as a reciprocal interaction between person, behaviour, and environmental factors [40, 41]. A central construct in SCT is self-efficacy, a person’s belief in his or her ability to successfully perform a certain action. In this programme we targeted parental self-efficacy (PSE), which refers to a parents’ belief in his or her own ability to perform specific action for example serving vegetables to each meal eaten at home. Other important constructs in the SCT, relevant for our intervention, are observational learning, behavioural capability, outcome expectations and self-regulation. In this programme PSE, parental knowledge, attitude, preference, care and control, role modelling and willingness to change were identified as potential mediators of change regarding children’s dietary and physical activity habits and weight development. Based on this analysis, three intervention components were defined and materials developed according to the steps of intervention research relevant to this study [42]. The intervention components were: 1) A brochure with health information targeting parental knowledge; 2) Motivational interviewing targeting parental self-efficacy, willingness to change and care and control; and 3) classroom activities targeting children’s knowledge, attitudes and preferences and indirectly parental role modelling. A study protocol has been published describing this in more detail [37].

### Intervention components

#### Health information

A brochure was developed based on a literature review [43] with the aim of increasing parental knowledge regarding how to promote children’s healthy dietary and physical activity habits. The brochure contains facts and advice for parents within seven areas: 1) parental feeding practices; 2) healthy food and family meal times; 3) physical activity; 4) sweets, snacks, ice-cream and soft drinks; 5) fruit and vegetables; 6) physical inactivity, screen time, and commercials; 7) sleep. The brochure is written in basic, easy-to-read Swedish with many illustrations. The brochure was translated into Arabic and Somali, the two most common languages in the intervention area and checked by two Arabic and Somali speaking persons and thereafter revised. The brochure was sent home to parents. Group meetings were offered once at each school, giving parents an opportunity to discuss the content of the brochure with two research assistants.

#### Motivational interviewing

MI was used to target PSE to support healthy eating and physical activity for the child, parental care and control, and stimulate parental willingness to change. MI is a client-centred, goal-oriented communication style designed to strengthen personal motivation for a specific behaviour change [29]. In MI, self-efficacy is a central part of motivation and is therefore focusing on ensuring that a person has high self-efficacy for changing a specific behaviour.

Parents in the intervention group were offered two individual sessions of MI without the presence of the child. Two MI counsellors performed the sessions, which lasted for approximately 45 min. The parents met the same MI counsellor both times. During the first MI session, the parents used an agenda-setting tool to choose a target behaviour involving their child’s diet or physical activity that they wanted to change. This behaviour was subsequently explored together with the MI counsellor and the parent set a goal related to the target behaviour to work on at home until the second MI session, which served as a follow-up. The second session was offered either face-to-face or over the telephone at the parent’s choice. The MI sessions provide the most intense parental support and are therefore hypothesised to be the component of the Healthy School Start intervention with greatest impact on outcome.

#### Classroom activities

The aim of the classroom component was to increase children’s knowledge, influence their attitudes and preferences, and indirectly support parental role modelling. A teacher’s manual and a workbook for children were developed to facilitate the classroom activities, which were related to the different areas in the brochure sent home to parents. The materials were developed together with teachers and inspired by earlier school interventions [44, 45]. Before the start of the intervention, the workbooks were read and commented on by eleven parents of grade 1 children in two of the participating schools. The manuals were tested by four teachers in the participating schools.

The children were exposed to ten 30-min teacher-led sessions. The teachers were provided with a tool-box containing culturally appropriate images of common food products such as baklava, olives and bulgur wheat, and used the teaching manual for each session. After most sessions, the children were given homework to discuss and complete together with their parents. Back in the classroom, the teachers and children summarised the homework, so that each theme was repeated.

Control classes were offered the entire programme after the follow-up measurements were completed.

### Implementation strategies

The following implementation strategies were used:

*Schools:* The principals in each school signed a contract with the research group specifying the obligations and commitments of the schools and the research team.

*MI counsellors:* The two MI counsellors had training and professional experience of MI and met a satisfactory level of MI competence according to the Motivational Interviewing Treatment Integrity (MITI 3.0) Code [46] at the time of recruitment. The counsellors received additional training and were supervised nine times during the intervention by a member of the Motivational Interviewing Network of Trainers and part of the research team (ÅN). Supervision focused on the counsellors’ experiences and difficulties when performing MI with the parents and also included systematic feedback and MITI-coding on audiotaped MI sessions.

*Teachers:* The research team trained the teachers for the classroom activities for two hours before the start of the intervention. In addition, teachers were given continuous support by the research team through face-to face contact, telephone and e-mail throughout the intervention.

*Parents:* Parents were informed about the project at the first regular information meeting in school. When the intervention had started, an additional meeting was offered at each school for parents in the intervention group, where they could discuss issues related to diet and physical activity.

### Outcome evaluation

Data were collected at baseline between August and September 2012 (T1), directly after the intervention between April and May 2013 (T2) and at follow-up five months after the intervention, between September and October 2013 (T3).

#### Physical activity by accelerometry

Physical activity was objectively assessed using accelerometry (GT3X+, Actigraph, LCC, Pensacola, USA). This method has been used in many studies to measure physical activity in children and is considered valid and reliable [47, 48]. The accelerometers were worn on a belt at the right hip for seven consecutive days. The children were instructed to wear the monitors during waking hours and to remove them for activities involving water.

The software ActiLife Data Analysis, version 6.5.2., was used to analyse the accelerometer data. Physical activity was assessed between 7 am and 9 pm and was calculated for the whole week and during weekends. Children who provided at least 500 min of activity registration per day for a minimum of two days, including at least one weekend day, were included in the analyses. Data were defined as non-wear time and excluded if sequences showed 10 or more consecutive minutes of zero counts. The epoch length was set to 15 s. The threshold for sedentary intensity was defined as all activity below 100 cpm [49, 50], moderate to vigorous intensity was defined as all activity above 2000 cpm. The threshold for moderate intensity corresponds to a walking pace of about 4 km/h (3 METS) in children [51].

#### Health behaviours by parent report

Dietary indicators (fruit, vegetables and energy-dense products), physical activity habits, sedentary behaviour and sleep were measured through a validated parent-proxy questionnaire, the Eating and Physical Activity Questionnaire (EPAQ) [52], which was translated into Swedish and to some extent adapted to a Swedish context. Parents were asked to recall the child’s intake of selected indicator foods (snacks, sweets/chocolate, ice-cream, cakes/buns/cookies, fruits, vegetables, soft drink, flavoured milk and fruit juice) the previous weekday. For each food item the parent marked the number of servings on a scale with 7 categories: 0, 0.5, 1, 2, 3, 4, 5. For drink items the categories were: 0, 1, 2, 3, 4, 5, 6 or more servings. Servings were defined as: drinks = 1.5 dl, vegetables = e.g. 2 dl grated carrots/cabbage or a big tomato or 2–3 broccoli stalks, fruit = e.g. a small apple or about 10 grapes, snacks = 1.5 dl crisps or cheese doodles, sweets = about 1.5 dl of sweets or 4 pieces from a chocolate bar, cakes = a small bun or 5 small biscuits, ice-cream = a small popsicle stick or 1 dl ice-cream. The questionnaire has previously been validated against 24-h dietary recall in two to five-year-old children and showed significant Spearman rank correlations for different items ranging from 0.57 to 0.88 [52]. The Swedish language in the questionnaire was pre-tested by eleven parents of grade 1 children in two of the participating schools and the language was simplified where necessary. The EPAQ was distributed by mail to the parents and only available in Swedish. Parents were offered help to fill in the questionnaires on one occasion at each school.

#### Anthropometry

Height, weight and waist circumference measurements were performed in schools by two trained research assistants according to standardised procedures [37]. BMI was calculated as weight (kg) divided by height (m) squared. Overweight and obesity were defined according to the International Obesity Task Force recommendations [53]. BMI standard deviation score (BMI sds) was calculated according to a Swedish reference standard [54].

#### Socio-economic status

Area of residence and parental educational level were used as indicators of SES [55, 56]. The study was conducted in three areas in Stockholm County with low employment and low educational level. These areas are also targeted specifically by the government to support socio-economic development [57]. Parental educational level was self-reported and the highest level of education attained by either of the parents was used as an indicator of SES. The variable was dichotomised with low education corresponding to primary and secondary school (≤12 years of schooling) and high (>12 years of schooling) corresponding to third level education.

#### Region of birth

Parents were also asked to indicate their region of birth as “Sweden/the Nordic region”, “Europe” or “Outside Europe”. It was also possible to specify the country.

### Process evaluation

The process of the intervention was assessed as the dose the parents had received and the dose the teachers had delivered. Parents were asked at the first MI session if they had read the brochure. Teachers were asked whether they had completed the teaching sessions and workbooks and also documented how much time they had spent on each session. Fidelity to MI was measured through coding of sessions. All MI sessions were audio-recorded and each counsellor had eight sessions coded according to MITI 3.0 [46] by reliable coders at a university coding lab (MIC-Lab, Stockholm). Mean values on the MI behaviours measured by the MITI were calculated for each MI counsellor and compared to threshold values for acceptable delivery of MI.

The teachers in the control classes were continuously asked to document if they had engaged in any organised healthy lifestyle activities during the intervention, in order to monitor possible contamination.

### Statistical analyses

Descriptive data at baseline were analysed using the SPSS 22.0 software package (Chicago, Illinois, USA). Significance-testing for differences between intervention and control group at baseline was performed through independent sample *t*-test or Chi-square/Mann-Whitney *U*-Test. All children who had agreed to participate in the intervention were included in the analysis on an intention to treat basis. Analyses were also performed per protocol where families participating in one or two MI session were compared to the control group.

Prior to analyses, half-servings of the dietary indicators were slightly modified to fit a Poisson distribution by merging half-servings with single-servings. A sensitivity analysis was performed where half-servings were merged with zero-servings, with no differences in results. We also created aggregated variables indicating unhealthy foods (snacks, sweets/chocolate, ice-cream, cakes/buns/cookies), healthy foods (fruit and vegetables) and unhealthy drinks (soft drink, flavoured milk and fruit juice above one serving) to analyse food patterns. When aggregating the separate variables we used the original values, containing half-servings, in order to retain as much information as possible. After aggregation, an adjustment of the half-servings was made by merging them with the closest upper discrete value. Sensitivity assessment was performed by merging half-servings with the closest lower discrete value, which gave similar results.

School class was used as unit of randomisation and therefore Mixed-effect Regression analyses [58] with two levels (individual and school class) were undertaken to estimate the intervention effects. For continuous outcomes (total physical activity during the week and weekend (TPA), time spent in moderate to vigorous physical activity during the week and weekend (MVPA), time spent sedentary during the week and weekend, screen time, BMI sds) Mixed Linear Regression was performed. For count outcomes (servings of juice, soft drink, flavoured milk, vegetables, snacks, fruits, sweets, cakes and ice-cream consumed on the previous day) Mixed Poisson Regression was performed. The statistical software MLwiN (version 2.31, 2014, Bristol University) was used. Level of significance was set to p < 0.05.

In order to detect significant changes between the groups after the intervention and to see if the changes were maintained at follow-up we compared the measurement at baseline (T1) to the measurement after the intervention (T2) and then compared T1 with the follow up measurement (T3). At the latter analysis, we excluded those individuals who did not have values at T2 in order to perform both comparisons (T1-T2 and T1-T3) on the same individuals.

We first tested a crude model for all outcomes at T2 or T3 with group as the predictor and adjusted for baseline values of the relevant outcome. In a second step, sex and parental education were added to the model. Interaction between group and sex or group and parental education were tested and analyses were stratified if significant interaction terms were found. Regarding physical activity (TPA, MVPA and time spent sedentary) outcomes were also adjusted for accelerometer monitor wear time. In the case of sedentary outcomes (week and weekend) the outcomes were adjusted for MVPA during the same period [59]. Since 80.4 % of the parents were born outside the Nordic region, we also tested the models by adjusting for region of birth (a dichotomous variable: Nordic/outside Nordic). Region of birth was not significant in any of the analyses and was therefore not used in the final models. Lastly, a random intercept for school class clustering was tested to detect differences between the clusters. To assess the fit of the model, we compared −2 Log Likelihood values between the model with fixed main effects and the model where the random intercept was included. A sensitivity analysis was undertaken for significant outcomes (unhealthy foods and unhealthy drinks, cakes/buns/cookies, and BMI sds) where baseline values were imputed for missing data at T2 or T3.

The power calculation for this study was based on the assumption of an average 20 % increase of physical activity assessed by accelerometry in the intervention group. The estimated sample size was calculated for a two-sided test with the significance level of 0.05 and power was set to 90 % using a sample size calculator for cluster randomised trials [60]. The calculation showed that 12 school classes with a participation rate of 60 % in each class, approximately 144 children in total, were needed to detect a 20 % increase in physical activity between the intervention and control groups.

## Results

Ten children dropped out of the study (intervention: 4, control: 6). These children had weight status and parental education levels similar to the remaining sample.

Families classified as having low parental education comprised 47.1 % of the total sample. Of all the parents, 80.4 % were born outside of the Nordic region with Iraq, Eritrea, Somalia, Iran and Turkey as the most common countries of birth.

Table 1 shows descriptive data and results of independent sample *t*-test and Chi-square-test at baseline. There were no significant baseline differences between the groups, except for intake of ice-cream, chocolate and sweets; with children in the control group consuming significantly more than those in the intervention group. Also, the proportion of parents born outside of the Nordic region was higher in the control group.

**Table 1:** Descriptive characteristics of children at baseline categorised by intervention and control group

	Total	Intervention	Control	*p*	*n*
	*n* = 378	*n* = 185 (89 boys/96 girls)	*n* = 193 (98 boys/95 girls)		
	Mean (SD)	Mean (SD)	Mean (SD)		
Age (years)	6.3 (0.3)	6.3 (0.3)	6.3 (0.3)	0.84	378
Parental low education per family (%)	47.1	43.8	50.3	0.18	345
Parents born outside the Nordic region (%)	80.4	76.5	84.2	0.01	699
Anthropometry					
Weight (kg)	24.5 (5.0)	24.2 (5.0)	24.7 (5.0)	0.33	378
Height (cm)	120.3 (5.4)	119.9 (5.1)	120.6 (5.7)	0.23	378
Waist circumference (cm)	56.2 (5.7)	56.1 (5.7)	56.6 (5.7)	0.44	378
Body mass index (kg/m2)	16.8 (2.5)	16.7 (2.4)	16.9 (2.5)	0.51	378
BMI sds^a^	0.66 (1.37)	0.62 (1.33)	0.69 (1.42)	0.60	378
Normal weight^b^ (%)	67.5	68.6	66.3	0.63	378
Overweight and obese^b^ (%)	26.5	25.4	27.5	0.65	378
Underweight^b^ (%)	6.1	5.9	6.2	0.91	378
Physical activity					
TPA, all week (cpm)	775 (192)	792 (218)	758 (160)	0.11	327
TPA, weekend (cpm)	639 (207)	627 (211)	651 (204)	0.34	268
MVPA, all week (minutes)	89 (24)	89 (25)	88 (23)	0.82	327
MVPA, weekend (minutes)	66 (27)	64 (27)	69 (26)	0.15	268
Sedentary, all week (minutes)	322 (45)	318 (48)	326 (42)	0.08	327
Sedentary, weekends (minutes)	326 (64)	327 (65)	325 (64)	0.71	268
Child taken to playground etc in the past week (times/week)	2.04 (1.27)	2.00 (1.27)	2.09 (1.29)	0.57	291
Television/computer time (minutes/day)	128 (75)	124 (77)	133 (72)	0.34	301
Diet (servings the previous day)					
Fruit juice	0.57 (0.66)	0.56 (0.69)	0.57 (0.62)	0.72	253
Soft drink	0.29 (0.52)	0.26 (0.49)	0.32 (0.54)	0.32	232
Milk	1.21 (0.78)	1.16 (0.76)	1.26 (0.80)	0.18	280
Flavoured milk	0.33 (0.56)	0.28 (0.45)	0.38 (0.65)	0.36	227
Vegetables	1.03 (0.77)	0.95 (0.76)	1.11 (0.79)	0.28	277
Fruits	1.62 (0.96)	1.48 (0.85)	1.76 (1.05)	0.14	294
Snacks (crisps and cheese doodles)	0.31 (0.61)	0.27 (0.52)	0.36 (0.68)	0.26	263
Chocolate/sweets	0.47 (0.68)	0.36 (0.62)	0.57 (0.72)	0.05	276
Ice-cream	0.49 (0.76)	0.35 (0.61)	0.64 (0.87)	0.03	281
Cake/buns/cookies	0.57 (0.79)	0.51 (0.61)	0.64 (0.80)	0.43	274

*p* = between intervention and control groups

*BMI sds* body mass index standard deviation score, *TPA* total physical activity, *cpm* counts per minute, *MVPA* moderate to vigorous physical activity

^a^Defined according to Karlberg et al. 2001

^b^Defined according to Cole et al. 2012

### Diet

The parental response rate to the questionnaire measuring dietary intake ranged from 78 to 60 % for the different items at baseline, between 75 and 59 % at T2 and between 72 and 59 % at T3. At baseline 70 % of the participating children consumed at least 2 servings of fruit and vegetables daily at home. Forty percent of the children consumed at least one serving of unhealthy foods daily, at baseline.

Regarding intake of indicator foods, we found significant intervention effects on outcomes related to intake of unhealthy foods and drinks (Table 2). At T2 the intervention group had a significantly lower intake of unhealthy foods (aggregated variable: snacks, ice-cream, cookies and sweets) (*p* = 0.01). This effect was sustained in boys at T3 (*p* = 0.03). Intake of unhealthy drinks (aggregated variable: soft drink, flavoured milk and fruit juice above 1 serving) at T2 was significantly lower in the intervention group (*p* = 0.01) compared to the control group. This effect was not sustained at T3.

**Table 2:** Effects of intervention on dietary intake of indicator foods at T2 and T3

			T2					T3		
Servings^a^ the previous weekday	*n*	*b*	*p*	95 % CI	Between school class variance σ_u_^2^ (s.e.)	*n*	*b*	*p*	95 % CI	Between school class variance σ_u_^2^ (s.e.)
Separate variables										
Fruit juice	190	−0.24	0.16	−0.09 to 0.56	0.19 (0.23)	154	−0.09	0.70	−0.53 to 0.36	0.00 (0.00)
Soft drink/sugar syrup	162	−0.28	0.25	−0.76 to 0.19	0.00 (0.00)	126	0.02	0.95	−0.64 to 0.68	0.00 (0.00)
Flavoured milk	161	−0.47	0.15	−1.11 to 0.16	0.00 (0.00)	131	−0.04	0.92	−0.76 to 0.68	0.00 (0.00)
Vegetables	226	0.15	0.22	−0.09 to 0.38	0.00 (0.00)	196	0.02	0.85	−0.22 to 0.27	0.00 (0.00)
Snacks	195	−0.57	0.08	−1.19 to 0.06	0.00 (0.00)	162	−0.46	0.19	−1.16 to 0.24	1.35 (0.49)
Fruits	241	−0.15	0.13	−0.35 to 0.04	0.00 (0.00)	206	0.03	0.76	−0.18 to 0.25	0.00 (0.00)
Sweets/chocolate	210	−0.38	0.10	−0.82 to 0.07	0.00 (0.00)	173	−0.26	0.29	−0.73 to 0.21	0.00 (0.00)
Cakes/buns/cookies	212	0.00	1.00	−0.51 to 0.51	0.00 (0.00)	179	−0.33	0.12	−0.74 to 0.89	0.00 (0.00)
Girls^b^	104	−0.04	0.88	−0.55 to 0.47	0.00 (0.00)					
Boys^b^	108	−0.95	0.003	−1.58 to -0.32	0.00 (0.00)					
Ice-cream	222	−0.22	0.22	−0.57 to 0.13	0.00 (0.00)	186	−0.22	0.30	−0.65 to 0.20	0.00 (0.00)
Aggregated variables^c^										
Unhealthy food	230	−0.32	0.01	−0.56 to -0.07	0.19 (0.07)	198	−0.15	0.42	−0.51 to 0.22	0.95 (0.16)
Girls^b^						101	0.19	0.43	−0.28 to 0.67	0.79 (0.19)
Boys^b^						97	−0.50	0.03	−0.94 to -0.06	0.37 (0.14)
Unhealthy drink	214	−0.51	0.01	−0.90 to -0.11	0.26 (0.16)	182	0.05	0.83	−0.39 to 0.49	0.19 (0.20)
Healthy food	248	−0.02	0.79	−0.16 to 0.12	0.00 (0.00)	217	−0.03	0.68	−0.18 to 0.12	0.00 (0.00)

Results of Mixed Poisson Regression adjusted for sex, parental education and baseline value

*b* = Regression coefficient (beta), *p* = between intervention and control groups, CI = 95 % confidence interval

^a^Serving sizes (examples below)

Drinks = 1.5 dl

Vegetables = 2 dl grated carrots/cabbage or a big tomato or 2-3 broccoli stalks

Fruits = a small apple or a bunch of grapes (about 10)

Snacks = 1.5 dl of crisps or cheese doodles

Sweets = about 1.5 dl of sweets or 4 pieces from a chocolate bar

Cakes = a small bun or 5 small biscuits

Ice-cream = a small popsicle stick or 1 dl ice-cream

^b^Stratified analysis due to interaction effect (group × sex)

^c^Aggregated variables: unhealthy foods (snacks, sweets/chocolate, ice-cream, cakes/buns/cookies), healthy foods (fruit and vegetables) and unhealthy drinks (soft drink, flavoured milk and fruit juice above one serving)

Subjects are dependent observations between T1 and T2 and between T1 and T3

When each indicator food was assessed separately, an interaction effect with group and sex was found regarding intake of cakes/buns/cookies: boys in the intervention group had significantly lower intake at T2 compared to boys in the control group (*p* = 0.003). The differences were no longer significant at T3.

We noted a trend towards lower intake of all separate unhealthy food and drink outcomes in the intervention at T2, although none of them were significant. This trend was still visible at T3 for unhealthy foods but not for unhealthy drinks.

Regarding intake of healthy foods (aggregated variable: fruit and vegetables) we saw no significant differences between intervention and control groups either on the aggregated variable or the separate outcomes for fruit and vegetables.

All effects regarding intake of indicator foods at T2, unhealthy foods (aggregated variable), unhealthy drinks (aggregated variable), as well as the interaction effect on boys regarding cakes/buns/cookies (separate variable), were sustained in the sensitivity analyses after imputation of missing values. The same was true for the interaction effect on boys’ intake of unhealthy foods (aggregated variable) at T3.

No interaction effects between group and parental education were found for any of the dietary outcomes.

### Physical activity

The number of children who fulfilled the required level of at least 2 days (including one weekend day) of valid accelerometer data was 327 at baseline, 294 at T2 and 290 at T3. At baseline 290 (89 %) children reached 60 min of MVPA.

As shown in Table 3, no significant intervention effect was detected on any of the measurements of physical activity (TPA, MVPA, time spent sedentary and screen time) at T2.

**Table 3:** Effects of the intervention on physical activity levels at T2 and T3

		T2					T3			
	*n*	*b*	*p*	95 % CI	Between school class variance σ_u_^2^ (s.e.)	*n*	*b*	*p*	95 % CI	Between school class variance σ_u_^2^ (s.e.)
TPA, all week (cpm)^b^	189	−30.1	0.18	−74.0 to 13.7	0.00 (0.00)	150	−34.8	0.13	−79.3 to 9.7	2598.1 (9176.8)
TPA, weekends (cpm)^b^	189	−40.5	0.25	−110.2 to 29.2	0.00 (0.00)	150	−30.2	0.37	−96.1 to 35.6	0.00 (0.00)
MVPA, all week (minutes)^b^	189	−1.5	0.55	−6.6 to 3.5	0.00 (0.00)	150	−3.6	0.19	−8.9 to 1.8	41.2 (130.0)
MVPA, weekends (minutes)^b^	189	−0.6	0.88	−8.0 to 6.9	20.3 (186.3)	150	−3.2	0.45	−11.4 to 5.0	0.00 (0.00)
Sedentary, all week (minutes)^c^	189	1.5	0.68	−5.7 to 8.7	219.7 (150.2)	150	−9.2	0.03	−17.7 to -0.7	448.0 (165.2)
Sedentary, weekends (minutes)^c^	189	9.2	0.09	−1.4 to 19.9	610.4 (294.4)	150	−11.3	0.04	−22.3 to -0.4	434.2 (256.9)
Screen time min/day^a^	251	−2.6	0.79	−21.0 to 15.9	4443.5 (583.2)	222	−16.5	0.10	−36.0 to 3.0	1552.4 (2355.4)

Results of Mixed Linear Regression adjusted for ^a^sex, parental education, baseline value, ^b^monitor wear time ^c^and MVPA

*b* = Regression coefficient (beta), *p* = between intervention and control groups, CI = 95 % confidence interval

*TPA* total physical activity, *cpm* counts per minute, *MVPA* moderate to vigorous physical activity

Subjects are dependent observations between T1 and T2 and between T1 and T3

At T3, significant intervention effects were detected in terms of time spent sedentary: the intervention group was sedentary 9.2 min less during the entire week (*p* = 0.03) and 11.3 min less during the weekend (*p* = 0.04). However, these effects were not sustained in the sensitivity analysis.

No interaction effects between group and parental education or group and sex were found for any of the physical activity outcomes.

### BMI

Measurements on height, weight and waist circumference were performed on all (378) children at baseline, 359 at T2 and 345 at T3. There were no significant differences in BMI status in drop-outs between intervention and control group.

No significant intervention effect was detected for BMI sds at T2 or T3 (Table 4). However, a stratified analysis on weight status showed a significant intervention effect on children categorised as obese at baseline (*n* = 41). Children in the intervention group had significantly lower BMI sds (-0.21 BMI sds) at T2 (*p* = 0.03) compared to obese children in the control group. This effect was stable in the sensitivity analysis but not sustained at T3 although the direction was still negative.

**Table 4:** Effects of the intervention on BMI sds at T2 and T3

		T2					T3			
	*n*	*b*	*p*	95 % CI	Between school class variance σ_u_^2^ (s.e.)	*n*	*b*	*p*	95 % CI	Between school class variance σ_u_^2^ (s.e.)
BMI sds^a^	332	−0.03	0.46	−0.1 to 0.1	0.00 (0.00)	318	0.013	0.79	−0.1 to 0.1	0.08 (0.04)
BMI sds^a^ in overweight/obese^b^ children at T1	84	−0.02	0.75	−0.2 to 0.1	0.02 (0.10)	82	0.02	0.85	−0.2 to 0.2	0.14 (0.02)
BMI sds^a^ in overweight^b^ children at T1	47	0.12	0.23	−0.1 to 0.3	0.11 (0.02)	47	0.13	0.22	−0.1 to 0.3	0.13 (0.03)
BMI sds^a^ in obese^b^ children at T1	37	−0.21	0.03	−0.4 to -0.02	0.00 (0.00)	35	−0.05	0.79	−0.4 to 0.3	0.26 (0.06)

Results of Mixed Linear Regression adjusted for sex, parental education and baseline value

*b* = Regression coefficient (beta), *p* = between intervention and control groups, CI = 95 % confidence interval

Subjects are dependent observations between T1 and T2 and between T1 and T3

^a^Defined according to Karlberg et al. 2001

^b^Defined according to Cole et al. 2012

No interaction effects between group and parental education or group and sex were found regarding BMI.

### Per protocol analyses

Analyses including only families participating in 1 (*n* = 147) or both MI sessions (*n* = 86) compared to the control group gave essentially the same results as presented above.

### Process evaluation

All parents who attended the first MI session said they had read the brochure. Eleven group meetings with parents were undertaken where 45 parents participated.

The first MI session was performed with 146 parents (79 %) of whom 65 % were mothers, 31 % were fathers and 4 % participated as a couple. In the second session, 86 of the initial 146 parents participated. The level of MI delivered by the MI counsellors was satisfactory and is presented in Table 5.

**Table 5:** Fidelity to MI during intervention

MI behaviour	Counsellor A	Counsellor B	Threshold for acceptable MI
Global rating, “MI spirit”^a^	3.67	3.62	3.5
Reflection to questions ratio^b^	3.0	1.9	1
Open questions (%)^c^	53	19	50
Complex reflections (%)^d^	45	58	40

MI consistent behaviour of the MI counsellor in relation to thresholds for acceptable MI

Values are means of MI counsellor behaviour during MI sessions

^a^Holistic evaluation of counsellors expression of MI spirit (equal collaboration with client about the change + evoking client speech about the change + supporting client’s autonomy regarding the change)

^b^Frequency count of counsellor behaviour regarding the specific MI technique reflections in relation to questions; ratio between the total number of questions and reflections stated by the counsellor

^c^Frequency count of counsellor behaviour regarding questions; percentage of open questions posed by the counsellor

^d^Frequency count of counsellor behaviour regarding the specific MI technique reflections; percentage of complex reflections stated by the counsellor

In the 13 classes which reported their work with the classroom component, teachers spent on average 33 min on each lesson, ranging from 20 to 150 min. Eleven classes performed all 10 lessons, 4 classes performed 9 lessons, and 1 class performed 8 lessons. Regarding the home assignments in the work book, 12 of the 16 intervention classes completed all 9 of the assignments, 1 class completed 8 assignments and 3 classes completed 1 to “a few” of the home assignments. However, the data do not show whether the assignments were completed at home, as intended, or at school.

As documented by teachers, control classes did not conduct any other organised healthy lifestyle activities in the control classes.

## Discussion

This study evaluated the effectiveness of the Healthy School Start parental support programme to promote healthy dietary habits and physical activity in children from families with low SES and a high proportion of foreign-born citizens. The results show an intervention effect on outcomes related to intake of unhealthy foods and drinks. There was no intervention effect on physical activity and BMI sds for the whole group. However, BMI sds decreased significantly among obese children. The decrease in consumption of unhealthy foods among boys was sustained at 5 months follow-up. Fidelity to the programme was satisfactory for all three intervention components although only 79 % of the parents participated in the first MI session and 47 % in the second.

### Diet

Our results showed a significantly decreased intake of unhealthy food and drinks by children in the intervention group. At follow-up, this effect was sustained for boys. No effect was found on fruit or vegetable intake. This may be explained by high intake at baseline, when 70 % of the children consumed at least two servings per day of fruit and vegetables at home, which is in line with official recommendations. As many as 40 % of the children reported a daily intake of unhealthy foods at baseline and it is therefore encouraging that the intervention was effective in targeting this problem. A systematic review of parental support interventions has confirmed that children’s dietary habits can be improved through parental counselling but that attrition is a problem in low SES groups [27].

Previous studies have used MI counselling with parents to improve children’s diet, but most have targeted overweight or obese children. In an Italian study, 372 families with overweight or obese children aged 4–7 years received six MI sessions delivered by pediatricians over one year. A non-significant reduction in unhealthy dietary intake (desserts, fried food, sweetened snacks/candy and sweetened drinks) in the intervention group was detected [61]. No monitoring of MI fidelity was reported in this study. The High Five for Kids trial [62] included 465 parents of overweight or obese two-to-seven-year-old children. The intervention group received four MI sessions delivered by nurses, and three additional supportive telephone calls over one year. There was a non-significant trend towards decreasing intake of fast food and sweetened beverages in the intervention group compared to the control group.

Both studies using MI counselling targeting overweight or obese children, included three or more MI sessions and families did not have low SES, whereas in our study all children were included of which about half of the families had a low educational background. Despite the difference regarding the target group, our results are similar to those previous studies, using MI, including our first trial of this programme where we also found an effect on diet but not on physical activity [36]. This leads us to conclude that MI with parents, together with information and practical activities, seems to be effective in improving children’s diet in areas of low SES and with a high proportion of foreign born citizens.

### Physical activity and sedentary behaviour

In this study we did not find significant intervention effects on any of the physical activity outcomes. This lack of effect is not surprising considering that at baseline 89 % of all children in the study were sufficiently active according to international recommendations, engaging in at least 60 min of MVPA per day. Other studies using individual counselling have shown contrasting results [63, 64]. In a Finnish study, parents received counselling once a year over three years and the results showed positive effects on physical activity [64]. In contrast, in another study, face-to-face counselling during 6 months in the home did not result in changes in physical activity or sedentary behaviour [63]. This lack of effect is in line with our study, which also had duration of six months.

Apart from the satisfactory level of physical activity among the children at baseline, the lack of effect could also be due to the short duration of the intervention. The reason for choosing a relatively short intervention period of six months was the waiting-list control design of the study, where the control classes were promised to receive the intervention after the follow-up measurements were finished one year later. Our results are in line with our systematic review [27] where we found that it is difficult to increase children’s physical activity through parental support programmes.

Studies using MI as the counselling method have shown contrasting results regarding physical activity. In a 5-month controlled study with nine-year-old children of which a majority had normal weight, parents received 3 MI sessions with additional telephone calls as part of a multicomponent intervention [65]. The intervention also included extra physical activity in school and activities for both children and parents, including group discussions of dietary habits and physical activity. Fidelity to MI was not monitored and physical activity was measured through self-report in interviews with parents. A significant increase in physical activity was reported in the intervention group compared to the control group. Similar results were found in two other studies which both targeted overweight and obese children in primary care [61, 66]. However, only one of the studies reported MI fidelity [66]. Another study using MI showed no significant differences in physical activity between intervention and control groups [62].

Thus, use of MI either as a single method or as part of a complex intervention, does not have equally convincing results on physical activity as on diet. The studies that did find a positive intervention effect were mostly obesity management studies, where children probably had relatively low activity levels at baseline.

### BMI

Our results showed no difference between the intervention and control groups for weight development after the intervention. However, among obese children (*n* = 41), BMI sds differed significantly between the intervention and control group post-intervention. Parental support interventions to prevent overweight and obesity can be effective [23, 26], especially if of high intensity and this is true even in groups with low SES [67–69]. However, non-participation and attrition is a problem to be addressed.

Several studies have used MI either as a single method or as part of a complex intervention, but again fidelity is seldom reported. The study by Centis et al [65], using MI counselling, showed a significant decrease in BMI sds between the intervention and the control group in mainly normal weight children. In addition to MI, parents received weekly telephone calls providing support and additional nutrition information, which probably facilitated the change processes. Several MI interventions targeting children with weight problems have failed to detect any significant decreases in BMI [61, 66, 70]. However, the High Five for Kids Trial [62] targeting overweight and obese children found positive intervention effects where BMI sds decreased among girls and children in families with low SES. The BMI2-trial involving 633 parents of overweight children aged 2–8 years found significantly lower BMI in the most intense MI group compared to controls at 2-years follow-up [71]. MI fidelity was monitored during MI training, but not during the intervention. In contrast, another trial in overweight or obese children using MI with parents and additional telephone support had no such effect [72]. However, this was a preliminary study only including 60 children which may explain the lack of significant effects. Therefore, the decrease in BMI sds seen in our study among overweight or obese children is encouraging, even though the effect was transient. We believe that increasing the duration of all the intervention components and the number of MI sessions might yield stronger and more sustainable effects.

### Strengths and weaknesses of the study

This study has several strengths. First, a study protocol has been published ahead of the intervention including a clear description of the intervention theory, components, proposed mediators and outcomes [37]. All materials were pre-tested and culturally adapted to the multinational diversity in the target group by translation into the two most common languages in the target group, Arabic and Somali, as well as adaptation regarding commonly used foods. Second, it has a high quality cluster-randomised controlled design, a relatively large sample size, and we used objective assessment of physical activity, sedentary behaviour and anthropometry. Third, we evaluated the process of all three intervention components, and found that it was satisfactory, although only 46 % of the parents participated in both MI sessions. Monitoring MI fidelity is particularly important, and this was done by reliable coders using a valid and reliable instrument.

The fact that MI was delivered by members of the research team with high MI competence constitutes a strength in terms of internal validity, but a limitation in terms of external validity, as school nurses do not presently have this competence. Another limitation was the parental questionnaire, including diet, which has not been validated for the specific target group. Self-report might lead to under reporting of unhealthy behaviours and over reporting of healthy behaviours due to social desirability. Furthermore, the children’s usual diets might have been captured more accurately if repeated recalls had been performed. We also faced a high proportion of missing values in questionnaire responses regarding dietary intake and screen time which may have biased the results. We performed a sensitivity analysis in order to account for this which however did not essentially change the results. There may also have been a selection bias in the sample of families, with low participation of families where Swedish is not spoken. Finally, even though we checked for contamination in control classes at school, we cannot exclude the possibility that contamination has taken place in the home environment, which would tend to weaken the effects of the programme.

### Implications for future research

Changes in dietary intake and weight development in children with obesity in our study were significant and comparable to other studies. The intervention showed no effect on physical activity outcomes. There are several lessons to be learnt from this and our previous evaluation [36] of the Healthy School Start programme. Since changes in behaviours and formation of habits can take a long time and effects are short-lived, the programme might benefit from being extended from pre-school class to the first year of school to get stronger intervention effects and to maintain the effects long-term. Regarding the MI component, reminders could perhaps be conducted in the form of telephone calls as done in other studies [62, 65, 66]. The programme might also have been more successful if focus had been on one specific behaviour. Furthermore, cultural diversity in diet and physical activity might have influenced the effect of the intervention. Also, in a prevention study similar to this one, parents often refused to label a concern about diet or physical activity as a problem, but rather as something needing a minor adjustment [73]. It might therefore be necessary to fine tune the use of MI according to the parent’s culturally based expectations on health communication and to their views of the target behaviour both when eliciting and when providing information with a preventive purpose. The process evaluation of the HSS programme with teachers and parents [74] suggests that a better tailoring of intervention components to participants’ needs and capabilities may increase engagement in the intervention, which could potentially lead to better outcomes.

Achieving sustainability of a programme like this one requires integration into school routines. Delivering the intervention through the school health care services could be suitable as they are an ideal structure for reaching all children and their families independent of social background.

## Conclusion

The Healthy School Start Study shows that it is possible to influence unhealthy dietary intake and weight development in children from families with low SES and a high proportion of foreign-born citizens by providing individual parental support in a school context. However, the positive effects were short-lived. Therefore, the programme probably needs to be prolonged and/or intensified in order to obtain stronger and more sustainable effects, which can be justified based on the principle of proportional universalism. This study may therefore be an important contribution to the further development of evidence-based parental support programmes to prevent overweight and obesity in children in disadvantaged areas.
